# Fatty acid profile and estimated desaturase activities in whole blood are associated with metabolic health

**DOI:** 10.1186/s12944-020-01282-y

**Published:** 2020-05-21

**Authors:** Karianne Svendsen, Thomas Olsen, Tove C. Nordstrand Rusvik, Stine M. Ulven, Kirsten B. Holven, Kjetil Retterstøl, Vibeke H. Telle-Hansen

**Affiliations:** 1 Department of Nutrition, Institute of Basic Medical Sciences, Faculty of Medicine, University of Oslo, Oslo, Norway; 2 The Lipid Clinic, Department of Endocrinology, Morbid Obesity and Preventive Medicine, Oslo University Hospital, Oslo, Norway; 3 National Advisory Unit on Familial Hypercholesterolemia, Department of Endocrinology, Morbid Obesity and Preventive Medicine, Oslo University Hospital, Oslo, Norway; 4 Faculty of Health Sciences, Oslo Metropolitan University, P.O. Box 4 St Olavs Plass, 0130 Oslo, Norway

**Keywords:** Fatty acids, Desaturases, Obesity, Metabolic disease, Stearoyl CoA desaturase, SCD1, D5D, D6D

## Abstract

**Background:**

The aim was to investigate if fatty acid profile and estimated desaturase activities; stearoyl CoA-desaturase (SCD), delta-5-desaturase and delta-6-desaturase (D5D; D6D), differ between individuals with metabolically healthy (MH) and unhealthy (MU) phenotypes. We also explored these associations according to BMI categories.

**Methods:**

Men and women at moderately elevated risk of cardiovascular disease were included in this cross-sectional study (*n* = 321). If subjects met ≥4 out of 5 criteria (elevated triglycerides, total and LDL-cholesterol, HbA1c and low HDL-cholesterol), they were classified as MU (*n* = 52). If levels were within reference ranges for ≥3 of the same criteria, subjects were classified as MH (*n* = 150). Utilizing the entire population, a score ranging from 0 to 5 denoting the number of MU criteria met was computed. Estimated desaturase activities were calculated as product-to-precursor ratio of fatty acids in whole blood (SCD16 [16:1n7/16:0], SCD18 [18:1n9/18:0], D5D [18:3n6/18:2n6], D6D [20:4n6/20:3n6]).

**Results:**

Individuals with MH had lower estimated SCD16 and SCD18 activities, whereas estimated D6D activity was higher compared to MU. Similar, SCD16 and SCD18 increased, whereas D6D decreased with increasing criteria of MU. Trends were similar across BMI categories.

**Conclusions:**

This study supports the notion of estimated desaturase activities as possible novel biomarkers of metabolic health irrespectively of BMI.

## Introduction

Cardiometabolic disorders, including type 2 diabetes (T2D) and cardiovascular disease (CVD) remain the major contributors to the global burden of disease [1]. Obesity is an important risk factor for cardiometabolic disorders, and is thus associated with insulin resistance, glucose intolerance and dyslipidemia [2]. However, not all obese individuals fit into this traditional phenotype, and a subgroup has been described with metabolically healthy (MH) obesity [3, 4]. Compared with metabolically unhealthy (MU) obesity, the MH obesity phenotype has a favorable lipid profile and a normal to slightly reduced insulin sensitivity, despite similar body mass index (BMI). Comparable patterns have been observed among individuals with overweight (BMI 25–30 kg/m^2^). Normal weight individuals (BMI 20–25 kg/m^2^) are generally considered MH, but MU normal weight individuals also exist [5, 6]. In an American population, about 2/3 were considered MU despite normal weight [7].

The fatty acid (FA) profile in the circulation is influenced by dietary and lifestyle factors [8, 9], and an unfavorable FA profile has been suggested to predict risk of T2D, CVD and the metabolic syndrome (MetS) [10–14]. Some FAs can be produced endogenously through de novo lipogenesis [15]. Stearoyl-CoA desaturase-1 (SCD1) is a rate-limiting enzyme in catalyzing the desaturation of monounsaturated fatty acids (MUFAs) from saturated fatty acids (SFAs). Delta-5-desaturase (D5D) and delta-6-desaturase (D6D) catalyze production of polyunsaturated fatty acids (PUFAs) (Table 1).

**Table 1:** Stearoyl-CoA desaturase-1 (SCD1), Delta-5-desaturase (D5D) and Delta-6-desaturase (D6D) with purpose, precursor, product and estimated desaturase activity

**Desaturase enzyme name**	**Purpose**	**Precursor**	**Product**	**Estimated desaturase activity**
**SCD1**	Desaturation of monounsaturated fatty acids from saturated fatty acids			
**SCD16**		C16:0 (*palmitic acid*)	C16:1n7 (*palmitoleic acid*)	16:1n7/16:0
**SCD18**		C18:0 (*stearic acid*)	C18:1n9 (*oleic acid*)	18:1n9/18:0
**D6D**	Desaturation of polyunsaturated fatty acids (PUFAs)	C18:2n6 (*linoleic acid*)	C18:3n6 (*γ-linolenic acid*)	18:3n6/18:2n6
**D5D**	Desaturation of polyunsaturated fatty acids (PUFAs)	C20:3n6 (*dihomo-γ-linolenic acid*)	C20:4n6 (*arachidonic acid*)	20:4n6/20:3n6

An altered FA profile with a high proportion of C16:0 (palmitic acid) increased estimated SCD1 and D6D activities and decreased levels of C18:2n6 FA (linoleic acid) and estimated D5D activity have been associated with insulin resistance, CVD, obesity and development of MetS [16, 17]. Particularly the positive correlation between SCD1 activity, insulin resistance and fat mass has been observed both in animal models and in large human cohorts where SCD1 activity is estimated as product-to-precursor ratios in blood [17–19]. However, it is not known whether alterations in plasma FA profile and desaturase activity is associated with metabolic phenotypes independently of body mass. Hence, the objective of this study was to advance our understanding of the role of FA profile and estimated desaturase activities for the metabolic risk profile (MH and MU) in otherwise healthy subjects over different BMI categories.

## Methods

### Study population

The population was obtained from the Vascular lifestyle-Intervention and Screening in phArmacies (VISA)-study previously described in detail [20]. In short, a subset of 1318 individuals were screened and 582 with moderately elevated CVD risk participated in an 8-week randomized controlled trial (RCT) of lifestyle changes [21]. Of the 582 participants, 321 individuals were followed up one year after the RCT, and were accordingly included in the present cross-sectional study.

Subjects were ≥ 18 years and had BMI between 18.5 and 44.4 kg/m^2^. Importantly, despite moderately elevated risk factors for CVD, all subjects were free of CVD and did not use any medications known to affect serum levels of lipids, glucose, blood pressure or inflammatory markers. Subjects were characterized according to metabolic health phenotypes based mainly on the criteria of Karelis et al. [22]. Subjects were considered MH when fulfilling at least three of the following five criteria: fasting triglycerides < 1.7 mmol/l or < 2.1 mmol/l non fasting, total cholesterol < 5.2 mmol/L, low density lipoprotein-cholesterol (LDL-C) < 2.6 mmol/L, high density lipoprotein-cholesterol (HDL-C) > 1.3 mmol/L and hemoglobin Ab1c (HbA1c) < 5.7%. Subjects were considered MU when fulfilling at least four out of the five following criteria: triglycerides ≥1.7 mmol/L or ≥ 2.1 mmol/L (if not fasting), total cholesterol ≥5.2 mmol/L, LDL-C ≥ 2.6 mmol/L, HDL-C ≤ 1.3 mmol/L and HbA1c ≥5.7%. Using measured weight and height, BMI was calculated as weight (kg) divided by the square of height (m^2^). We defined normal weight, overweight and obese (BMI ≥ 30 kg/m^2^), according to the definition by World Health Organization [23]. To make use of data from the entire population, we computed a score based on criteria for MU ranging from 0 to 5 depending on the number of criteria met.

### Anthropometric measures

Data collection was performed by pharmacy staff (pharmacists, technicians or nurses), in accordance with standard operational procedures [20]. Prior to the study, pharmacy staff completed a training program consisting of online e-learning courses and practical training. Subjects were weighed in light clothing without shoes using a digital scale. Height was measured with standing, erect posture and feet against the wall with a wall mounted height board. Blood pressure was measured twice after resting for about five minutes, using A&D Medical blood pressure Monitor™ Model US767Plus30 [21].

### Biochemical measures

HbA1c, total cholesterol, HDL-C and triglycerides were measured by finger-pricks blood samples (Alere Afinion™AS100). At triglycerides > 7.34 mmol/L, HDL-C could not be measured. LDL-C was calculated using Friedewald’s formula. LDL-C was not calculated at triglycerides > 4.52 mmol/l. Non-HDL-C was calculated as total cholesterol – HDL-C [21].

### Fatty acid composition in whole blood and desaturase estimation

FA profile was obtained from whole blood by using dried blood spots (DBS) (VITAS™ Analytical Services) [24]. DBS is a bio-sampling method where capillary blood, obtained by a finger-prick lancet, are dripped onto spots on filter collection cards (DBS card) [25]. The completed DBS card dried in room temperature for two to four hours before it was stored in an aluminium bag at 1–4 degrees Celsius until shipment to VITAS (Oslo, Norway) for analysis. Fatty acids methyl esters (FAME) were extracted from whole blood (plasma and cells) [26], and were analysed using Gas Chromatography – Flame Ionization Detector (GC-FID) after direct transmethylation. Whole blood FA profile was given in % FAME. The desaturase activities were estimated as the product-to-precursor ratios of FAs in whole blood according to the following: SCD1 = C16:1n7/C16:0 and C18:1n9/C18:0, hereafter referred to as SCD16 and SCD18, respectively. D5D = C18:3n6/C18:2n6 and D6D = C20:4n6/C20:3n6 (Table 1).

### Questionnaires

From the validated, short food frequency questionnaire (FFQ), VISA-FFQ [27], we obtained data on intake of specific food groups and duration (minutes) and intensity (moderate and vigorous) of physical activity per week. Data on age, sex, smoking habits, marital status, ethnicity, educational level and income had been reported previously (− 52 weeks) through the baseline questionnaire [21].

### Statistical analysis

Baseline characteristics were given as means ± standard deviations (SD) or median and 25th and 75th percentiles for continuous variables, and counts (%) for categorical variables. T-tests were used to assess differences in continuous variables, whereas Chi-Square tests were used for categorical variables. Non-normal variables were log-transformed before analysis.

Differences in FA profiles and estimated desaturase activities between the MU and MH groups were assessed using linear regression models adjusted for age and gender. Values are reported as estimated marginal means and corresponding 95% confidence intervals (CI). Furthermore, the association of FA profiles and estimated desaturase activities with increasing numbers of criteria for MU were investigated using linear regression adjusted for age and gender. We further explored whether trends were similar in BMI categories by adding an interaction term to the models (BMI × metabolic status) to assess whether the association of metabolic status with FA profiles depended on BMI. Finally, we used principal component analysis (PCA) to explore whether clusters of whole blood FAs were associated with MH and MU. All individual FAs were selected and normalized (scaled and centred) prior to PCA. Scree plots were examined and revealed three components explaining ~ 52% of the total variance in the dataset after application of varimax rotation. We proceeded with principal component (PC) 1 and PC2 and the computed PC scores were scaled and centred and given as z-scores. The PCA procedure was performed using the psych package for R. Linear regression was used to assess differences in PC1 and PC2 between MU and MH. A *p*-value of < 0.05 was considered statistically significant. We opted to not adjust for multiplicity due to the exploratory nature of the study. Statistical analyses were carried out using IBM SPSS version 23 and R version 3.6.3, using packages included in the “tidyverse” package selection, “psych” and “princomp”. Plots were made with the ggplot2 package for R.

## Results

### Metabolic health, fatty acid profile and estimated desaturase activities

Characterization of the participants is presented in Table 2. Of 321 subjects, 37.1% (*n* = 119) were categorized as normal weight, 40.2% (*n* = 129) were overweight and 22.7% (*n* = 73) were categorized as obese. Of these, 202 subjects met the criteria for MH and MU, and were subsequently categorized as MH (*n* = 150) or MU (*n* = 52). The individuals with MH phenotype were slightly younger (55.5 ± 14.8 years vs. 60.5 ± 12.1 years) and reported higher physical activity (median [25, 75th percentile]: 231 (79.3, 469) minutes vs. 114 (0, 247) minutes) than individuals with MU phenotype. There were no differences in blood pressure, gender, years of education, or prevalence of smokers between MH and MU subjects (Table 2).

**Table 2:** Characteristics of metabolically healthy and unhealthy subjects

	**Metabolically healthy (*n* = 150)**	**Metabolically unhealthy (*n* = 52)**	***P***
**Demographics**			
Age, years (mean ± (SD))	55.5 ± 14.8	60.5 ± 12.1	0.017
Men, n (%)	34 (22.7)	19 (36.5)	0.132 ^1^
≤ 13 years of schooling, n (%)	77 (54.6)	26 (51.0)	0.854 ^1^
Smokers (daily or occasional), n (%)	18 (12.3)	5 (9.6)	0.607 ^1^
**Risk factors** (mean ± (SD))			
BMI, kg/m^2^	26.6 ± 4.6	28.8 ± 4.5	0.003
Total cholesterol, mmol/l	6.2 ± 1.1	6.7 ± 1.0	0.012
LDL-cholesterol, mmol/l	3.8 ± 1.0	4.0 ± 0.9	0.073
HDL-cholesterol, mmol/l	1.9 ± 0.5	1.4 ± 0.4	< 0.001
NonHDL, mmol/l	4.3 ± 0.9	5.2 ± 0.9	< 0.001
Triglycerides, mmol/l	1.3 ± 0.5	2.9 ± 1.1	< 0.001
HbA1c, %	5.4 ± 0.2	5.8 ± 0.4	< 0.001
Systolic BP, mmHg	126.2 ± 16.1	126.6 ± 16.8	0.857
Diastolic BP, mmHg	79.5 ± 9.9	80.4 ± 10.1	0.567
Physical activity, min/week^2,3^	231 (79.3, 469)	114 (0, 247)	0.002

Demographics as frequency [n (%)] and age were obtained 52 weeks prior to the present data collection. Risk factors are expressed as mean ± SD. *P* values from Student’s t-test. *P* is significant at 0.05 level. ^1^*P* value from Chi-square test. BMI, body mass index; BP, blood pressure; HbA1c, glycated hemoglobin A1c. ^2^Given as median (25th – 75th percentile). ^3^Mann-Whitney U test

**Fig. 1:**
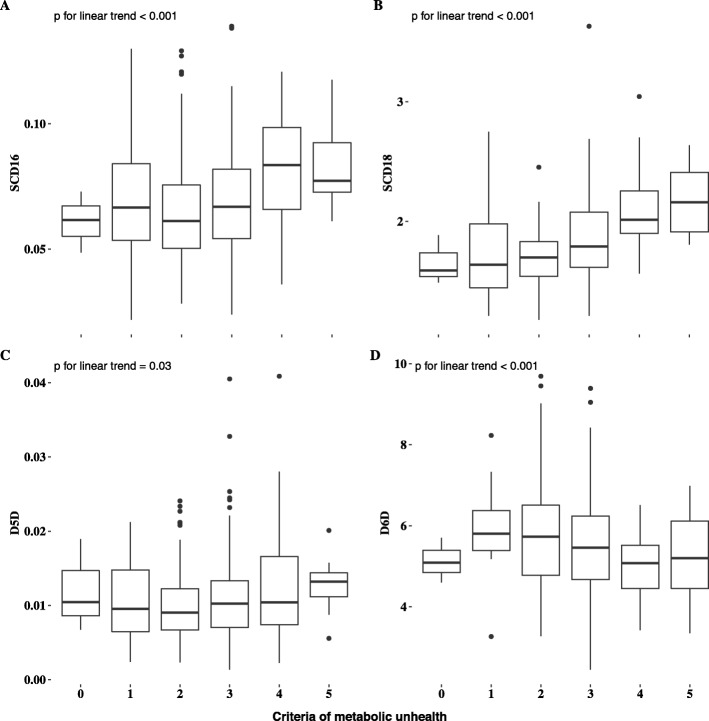
Boxplots showing **a**) SCD16, **b**) SCD18, **c**) D5D and **d**) D6D activity indices accross metabolic health criteria

Of the FAs measured, C18:0 was higher, whereas C14:0 and C16:0 were lower in the MH group compared with MU group. Furthermore, the MH group had lower levels of total MUFAs, C16:1n7, C18:1n9 and estimated SCD16 and SCD18 activity compared to the MU group (*P* < 0.001 for all). Except for C18:3n3, total n6 FA, n3 FA and D6D, but not D5D, were significantly higher in the MH group (Table 3). We observed that estimated SCD16 and SCD18 activities (*P* for both < 0.001) increased with increasing number of criteria for MU (ranging 1–5). A clear decrease was observed for estimated D6D activity (*P* < 0.001) and a slight decrease was observed for estimated D5D activity with increasing number of criteria for MU (*P* = 0.03) (Fig. 1).

**Table 3:** Fatty acid profile and estimated desaturase activity in metabolically healthy and unhealthy subjects

**Fatty acids, %FAME**	**Metabolically healthy (*n* = 150)**	**Metabolically unhealthy (*n* = 52)**	***P***
C12:0	0.12 (0.1, 0.14)	0.14 (0.12, 0.17)	0.169
C14:0	0.91 (0.85, 0.98)	1.16 (1.06, 1.25)	< 0.001
C15:0	0.25 (0.24, 0.26)	0.25 (0.23, 0.26)	0.658
C16:0	21.51 (21.25, 21.78)	22.35 (21.95, 22.76)	0.001
C17:0	0.35 (0.34, 0.36)	0.33 (0.32, 0.35)	0.049
C18:0	11.79 (11.62, 11.96)	10.98 (10.72, 11.24)	< 0.001
C20:0	0.09 (0.08, 0.09)	0.09 (0.09, 0.1)	0.167
SFA total	35.03 (34.72, 35.34)	35.3 (34.84, 35.77)	0.323
C16:1 n7	1.36 (1.26, 1.46)	1.72 (1.57, 1.86)	< 0.001
C18:1 n9	19.85 (19.45, 20.24)	22.51 (21.9, 23.11)	< 0.001
C18:1 c11	1.54 (1.49, 1.59)	1.51 (1.44, 1.59)	0.512
C20:1 n9	0.26 (0.24, 0.27)	0.24 (0.22, 0.26)	0.221
MUFA total	23 (22.55, 23.45)	25.98 (25.3, 26.66)	< 0.001
SCD16	0.06 (0.06, 0.07)	0.08 (0.07, 0.08)	< 0.001
SCD18	1.7 (1.64, 1.75)	2.08 (2, 2.16)	< 0.001
D5D	0.01 (0.01, 0.01)	0.01 (0.01, 0.01)	0.081
D6D	5.87 (5.65, 6.09)	5.14 (4.8, 5.48)	< 0.001
C18:2 n6	19.81 (19.34, 20.29)	18.94 (18.22, 19.66)	0.042
C18:3 n6	0.2 (0.18, 0.22)	0.22 (0.19, 0.24)	0.257
C18:3 n3	0.49 (0.46, 0.52)	0.61 (0.56, 0.66)	< 0.001
C20:3 n6	1.4 (1.35, 1.45)	1.38 (1.31, 1.46)	0.694
C20:4 n6	8.03 (7.77, 8.29)	7.01 (6.61, 7.4)	< 0.001
C20:5 n3	1.48 (1.33, 1.63)	1.06 (0.83, 1.29)	0.002
C22:5 n3	1.39 (1.34, 1.44)	1.21 (1.13, 1.28)	< 0.001
C22:6 n3	3.4 (3.25, 3.56)	2.98 (2.74, 3.21)	0.003
PUFA total	36.21 (35.63, 36.79)	33.4 (32.52, 34.28)	< 0.001
ω-3 total	6.77 (6.46, 7.08)	5.85 (5.37, 6.33)	0.001
ω-6 total	29.44 (28.91, 29.98)	27.55 (26.74, 28.35)	< 0.001

*P* values from linear regression models adjusted for age and sex. Values are estimated marginal means 95% confidence interval). *P* is significant at 0.05 level. SCD1, Stearoyl-CoA desaturase-1; D5D, delta-5-desaturase; D6D, delta-6-desaturase; SFA, saturated fatty acids; MUFA, monounsaturated fatty acids; PUFA, polyunsaturated fatty acids

### Principal component analyses

**Fig. 2:**
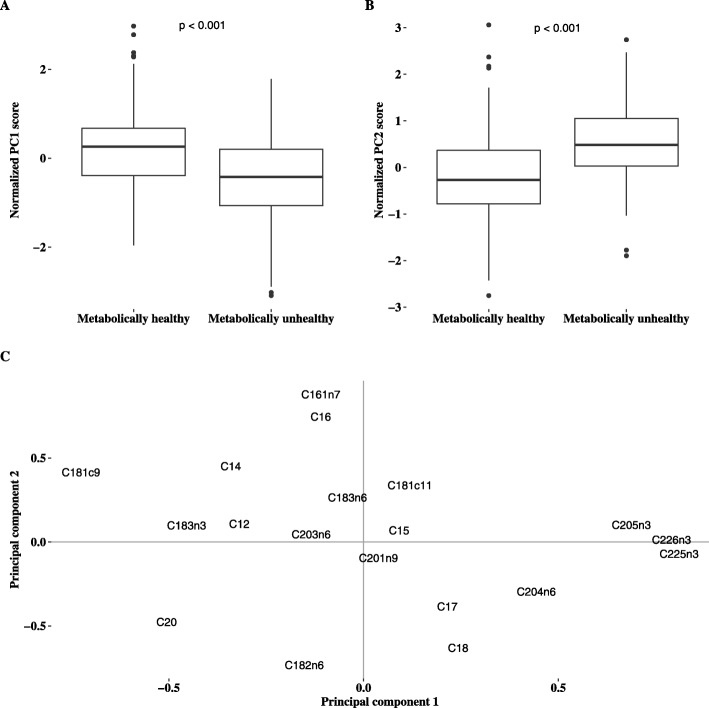
**a**) Boxplot for principal component 1 score, **b**) boxplot for principal component 2 score aand **c**) correlation between the individual fatty acids and prinicpal component 1 and 2

In order to explore whether clusters of FAs were different between the MH and MU groups, we performed PCA. All individual FAs were selected for analysis. PC1 and PC2 accounted for ~ 36% of the total variance in the dataset. Briefly, the FAs most positively correlated with PC1 were C20:5n3, C22:5n3 and C22:6n3 whereas C18:1n9 were strongly and inversely correlated with PC1. For PC2, the strongest positive correlations were observed for C16:0 and C16:1n7 and inverse correlations for C18:2n6 and C18:0. Compared with MH, individuals with MU had a lower mean z-score for PC1 and a higher mean z-score for PC2 as illustrated in Fig. 2.

### Association of metabolic health phenotypes and estimated desaturases by BMI category

We further investigated the MU phenotypes according to BMI categories. Characteristics of MH and MU individuals by BMI group are presented in Additional file 1.

Of the overall population of individuals with obesity (including those between MU and MH), 46.6% (*n* = 34) were categorized as MH (corresponding to 10.4% of the total population) and 23.3% (*n* = 17) as MU. The corresponding prevalence of MH among the individuals with overweight were 40.3% (*n* = 52) and 17.8% MU (*n* = 23). Among those with normal weight, 53.8% (*n* = 64) were MH and 9.2% (*n* = 11) were MU. Parameters were generally similar except for the metabolic status classification criteria. Individuals with MH and obesity reported higher physical activity with median (25th–75th percentile) 150 min (0, 362) per week compared with 38 min (0, 126) for MU with obesity (*P* = 0.031). The FA profile and estimated desaturase activities were similar between MU and MH subjects in each BMI category and no interactions were observed in the regression models (Additional file 2). The association between numbers of criteria met for MU overall showed the same patterns in each BMI category as for the total population (Additional file 3).

## Discussion

In a group of Norwegians with moderately elevated risk of CVD but non-medically treated, we found that estimated SCD16 and SCD18 activities increased with increasing number of criteria for MU, whereas estimated D5D and D6D activities appeared to decrease. Similar, some SFAs, total MUFA, estimated SCD16 and SCD18 were lower, whereas most PUFAs (both n3 and n6) and D6D were higher in MH than MU. These findings were also supported by results from PCA. Related findings were observed in subgroups according to BMI.

Findings from this study suggest that all of the investigated desaturases were related to metabolic health. The association between criteria for MU and desaturases was strongest for estimated SCD16 and SCD18 activities. In a subsample in the PREDIMED trial, estimated SCD16 but not SCD18 activity was increased in individuals with MetS [28]. Such an association may primarily be explained by obesity according to Warensjo et al. [12]. Additionally, findings from one large cohort study suggested that both SCD16 and SCD18 activities were associated with fat mass [18], further supporting that obesity can be a main driver of these associations. However, we observed associations between SCD activities and MU irrespectively of BMI subgroups. In line with our results, Lassale et al. found that individuals with a MU phenotype had higher risk of CVD than MH, irrespective of BMI [29]. One possible explanation for our findings could be different dietary intakes of SFAs, as estimated SCD16 activity has been shown to be higher after intervention with a diet high in SFA compared to a diet high in MUFA and PUFA [30, 31]. However, in the present study, we observed that intake of foods high in SFA did not differ between MH and MU (Additional file 4).

In addition to lower plasma levels of the SFAs C14:0 and C16:0, we also found lower levels of C16:1n7 and C18:1n9 in MH subjects, including MH obese compared with their MU counterparts. This finding corresponds to the lower levels of estimated SCD16 and SCD18 activities in MH, as endogen production of MUFAs is regulated by these desaturases [15].

MUFAs (C16:1 and 18:1) are major components of triglycerides, cholesterol esters and phospholipids, produced by de novo lipogenesis and carried in VLDL in the circulation [15]. This incorporation of MUFAs in VLDL, could partly explain the observed association between high levels of MUFAs and the MU phenotype, characterized by high levels of triglycerides, total cholesterol and LDL-C. Our findings are also supported by results from a NHANES survey where higher plasma concentration of SFAs and MUFAs were associated with increased levels of fasting plasma glucose and HbA1c [32]. The same pattern was found in the FINRISK study. Here, higher plasma levels of MUFAs in healthy, overweight subjects were associated with increased CVD risk, whereas higher levels of n6 FAs and docosahexaenoic acid (DHA) levels were associated with lower risk [33], in line with our study.

We found that the estimated activity of enzymes responsible for the desaturation of PUFAs, D6D and to some extent D5D activities, decreased with increasing numbers of criteria for MU. Both Zhao et al. and Mayneris-Perxachs et al. found that estimated D5D activity was inversely associated with metabolic dysregulation [28, 34], also in MH obese subjects [34]. The observed higher levels of estimated D6D in MH subjects in our study was therefore in contrast to other findings where D6D has been associated with increased risk of MetS and insulin resistance in individuals with overweight and obesity [28, 35]. The association has been suggested to be due to PUFA imbalance such as reduced n3/n6 FAs ratio [35]. However, the ratio in blood between n3 and n6 in the present study was not excessively high [35]. Neither where there any differences in dietary intake of fatty fish (high in n3 FA) between the MH and MU group.

Our findings suggests that SCD may influence the development of MU phenotype independently of fat mass and dietary intake. Consequently, other unmeasured metabolic markers could affect the association between SCD and MU phenotype. Genetics may play an important role, as several studies in rodents have shown that SCD16 deficient mice do not become overweight/obese or have metabolic disruptions even when fed a high fat diet [36]. In this regard, body fat distribution among subjects and across genders is another unmeasured factor that could have helped elucidated this results [37]. For instance, MH overweight and obese could have lower levels of visceral and ectopic fat [38, 39]. Furthermore, we observed MH to be more physically active than MU inn all BMI groups, indicating that lifestyle is likely to be important for metabolic health.

It is however possible that our study population is healthier than other populations. In total 10% defined as MH obese is among the highest prevalence independent of criteria used for metabolic health summarized by Liu et al. [40] and Wildman et al. [41]. Using BMI rather than waist circumference to define obesity often yields higher prevalence of MH obese [40]. Yet, other studies have found comparable prevalence of MH obese as the present study using ≥2 criteria for metabolic health [42]. Furthermore, subjects were enrolled in the present study based on background CVD risk and had previously attended an intervention study aiming at changing lifestyle behavior [21]. This might have influenced the study population and limiting the generalizability of results.

In line with this, we observed that in the present study, 9% (*n* = 13) of the MH were previously (1 year before) categorized as MU*.* This transition from MU to MH was due to favorable changes in diet, lifestyle and CVD risk factors previously described as long-term effects of participating in the VISA-study [21]. On another note, it has been suggested that MH overweight and obese phenotypes may be transient states, as individuals with MH overweight and obesity are still at increased risk of developing T2D and CVD compared to normal weight individuals [43]. FAs and desaturase activity indices could therefore be predictors of long-term development of metabolic disorders [12], such as a 10-year transition from MH to MU [34]. Hence, our results support that desaturases could be biomarkers of (future) metabolic health independent of BMI, although direction of association remains to be elucidated.

### Strengths and limitations

The main strength of the study is the well-characterized sample of 321 individuals free of disease but at moderately increased risk of CVD who were originally recruited from the general population [20, 21]. Limitations includes lack of generalizability, as the study population had been previously included in an intervention study aiming at improving health. Furthermore, we note that due to the cross-sectional nature of the study, the direction of the associations reported is impossible to infer and no information on causality can be obtained.

## Conclusion

We found that estimated SCD16 and SCD18 activities increased with increasing number of criteria for MU, and that estimated D6D activity and to some extent also D5D activity, decreased. The corresponding SFAs and PUFAs followed the patterns of the estimates activities of SCD16/SCD18 and D5D/D6D respectively. The findings persisted irrespectively of BMI. Hence, the study supports that these desaturases can be important novel biomarkers of metabolic health, although these analyses cannot answer whether the dysregulation of these desaturases are a cause or a consequence of lifestyle factors or genetics. This warrants further studies.

## Supplementary information

**Additional file 1.** Characteristics of metabolically healthy and unhealthy normal weight, overweight and obese subjects.

**Additional file 2.** Fatty acid profile and desaturase ratios in metabolically healthy and unhealthy normal weight, overweight and obese subjects.

**Additional file 3.** Association of criteria for metabolic health and estimated desaturase activities in BMI categories.

**Additional file 4.** Diet and lifestyle for metabolically healthy (MH) and unhealthy (MU) participants stratified by body mass index (BMI).

## Data Availability

The datasets used and/or analysed during the current study are available from the corresponding author on reasonable request.

## Abbreviations

CVD: Cardiovascular diseases
FA: Fatty acid
HDL: High density lipoprotein
HbA1c: Hemoglobin A1c
LDL: Low density lipoprotein
MetS: Metabolic syndrome
MH: Metabolic healthy
MU: Metabolic unhealthy
MUFA: Monounsaturated fatty acids
PUFA: Polyunsaturated fatty acids
SCD1: Stearoyl CoA-desaturase 1
SFA: Saturated fatty acids
T2D: Type 2 diabetes
