# Supplementary material for: Fatty acid profile and estimated desaturase activities in whole blood are associated with metabolic health
Source: Lipids Health Dis. 2020 May 21;19:102. doi: 10.1186/s12944-020-01282-y (PMC7243306; doi:10.1186/s12944-020-01282-y)
Supplement: Supplementary file 1 — Additional file 1. Characteristics of metabolically healthy and unhealthy normal weight, overweight and obese subjects. [file 12944_2020_1282_MOESM1_ESM.docx]

**Additional file 1:** *Characteristics of metabolically healthy and unhealthy normal weight, overweight and obese subjects.*

|  | Normal weight | | | Overweight | | | Obese | | |
| --- | --- | --- | --- | --- | --- | --- | --- | --- | --- |
|  | MH  (n=64) | MU  (n=11) | ***P*** | MH  (n=52) | MU  (n=23) | ***P*** | MH  (n=34) | MU  (n=18) | ***P*** |
| **Demographics, n (%)** |  |  |  |  |  |  |  |  |  |
| Men | 11 (17.2) | 4 (36.4) | 0.332 ^1^ | 19 (36.5) | 8 (34.8) | 0.348 ^1^ | 4 (11.8) | 7 (38.9) | 0.068 ^2^ |
| ≤ 13 years of schooling | 26 (42.6) | 5 (45.5) | 0.761 ^1^ | 28 (58.3) | 11 (50.0) | 0.789 ^1^ | 23 (71.9) | 10 (55.6) | 0.483 ^1^ |
| Smokers (daily or occational) | 9 (14.5) | 1 (9.1) | 0.890 ^1^ | 7 (14.0) | 2 (8.7) | 0.184 ^2^ | 2 (5.9) | 2 (9.5) | 0.754 ^2^ |
| **Risk factors** (mean, ± (SD)) |  |  |  |  |  |  |  |  |  |
| Age, years | 57.3 ± 14.9 | 64.0 ± 11.4 | 0.164 | 55.6 ± 14.6 | 63.9 ± 11.2 | 0.017 | 51.7 ± 14.4 | 53.9 ± 11.2 | 0.575 |
| BMI, kg/m^2^ | 22.7 ± 1.5 | 23.9 ± 0.8 | 0.001 | 27.1 ± 1.3 | 27.2 ± 1.4 | 0.636 | 33.3 ± 3.4 | 33.8 ± 3.6 | 0.654 |
| Total cholesterol, mmol/l | 6.4 ± 1.0 | 6.2 ± 0.9 | 0.461 | 6.1 ± 1.2 | 7.0 ± 1.1 | 0.002 | 6.0 ± 1.2 | 6.4 ± 0.9 | 0.148 |
| LDL-cholesterol, mmol/l | 3.8 ± 0.9 | 3.7 ± 1.1 | 0.806 | 3.8 ± 1.0 | 4.3 ± 1.0 | 0.068 | 3.7 ± 1.0 | 4.0 ± 0.8 | 0.342 |
| HDL-cholesterol, mmol/l | 2.1 ± 0.4 | 1.5 ± 0.4 | <0.001 | 1.7 ± 0.5 | 1.5 ± 0.5 | 0.059 | 1.7 ± 0.4 | 1.3 ± 0.2 | 0.001 |
| NonHDL-cholesterol, mmol/l | 4.3 ± 0.9 | 4.7 ± 0.9 | 0.282 | 4.4 ± 1.0 | 5.6 ± 1.0 | <0.001 | 4.3 ± 1.0 | 5.2 ± 0.8 | 0.002 |
| Triglycerides, mmol/l | 1.3 ± 0.5 | 3.0 ± 1.2 | 0.001 | 1.3 ± 0.6 | 3.2 ± 1.3 | <0.001 | 1.3 ± 0.4 | 2.6 ± 0.7 | <0.001 |
| HbA1c, % | 5.4 ± 0.2 | 5.7 ± 0.2 | <0.001 | 5.4 ± 0.2 | 5.7 ± 0.4 | <0.001 | 5.5 ± 0.2 | 5.9 ± 0.4 | 0.002 |
| Systolic blood pressure, mmHg | 124.9 ± 16.9 | 123.5 ± 16.2 | 0.799 | 128.7 ± 15.3 | 127.5 ± 19.1 | 0.774 | 124.7 ± 15.7 | 127.4 ± 14.7 | 0.548 |
| Diastolic blood pressure, mmHg | 75.3 ± 9.8 | 76.6 ± 10.6 | 0.690 | 82.9 ± 9.1 | 80.9 ± 11.7 | 0.419 | 82.1 ± 8.5 | 82.1 ± 7.0 | 0.982 |
| Physical activity, min/week^2,3^ | 247 (117, 508) | 152 (0, 319) | 0.117 | 218 (102, 469) | 160 (90, 393) | 0.369 | 150 (0, 362) | 38 (0, 126) | 0.031 |

Risk factors are expressed as mean ± SD. *P* values from Student’s t-test. *P* is significant at 0.05 level. ^1^*P* value from Chi-square test. MH, metabolically healthy; MU, metabolically unhealthy; BMI, body mass index; BP, blood pressure; HbA1c, glycated hemoglobin A1. ^2^Reported as median (25^th^-75^th^ percentile) ^3^*P*-value obtained from Mann-Whitney U test.
