# Supplementary material for: Fatty acid profile and estimated desaturase activities in whole blood are associated with metabolic health
Source: Lipids Health Dis. 2020 May 21;19:102. doi: 10.1186/s12944-020-01282-y (PMC7243306; doi:10.1186/s12944-020-01282-y)
Supplement: Supplementary file 2 — Additional file 2. Fatty acid profile and desaturase ratios in metabolically healthy and unhealthy normal weight, overweight and obese subjects. [file 12944_2020_1282_MOESM2_ESM.docx]

| **Additional file 2:** *Fatty acid profile and desaturase ratios in metabolically healthy and unhealthy normal weight, overweight and obese subjects* | | | | | | | | | |
| --- | --- | --- | --- | --- | --- | --- | --- | --- | --- |
|  | Normal weight | | | Overweight | | | Obese | | |
| Fatty acids | MH (n=64) | MU (n=11) | *P* | MH (n=52) | MU (n=23) | *P* | MH (n=34) | MU (n=17) | *P* |
| C12:0 | 0.11 (0.07, 0.14) | 0.11 (0.04, 0.18) | 0.994 | 0.13 (0.1, 0.16) | 0.17 (0.13, 0.22) | 0.104 | 0.1 (0.07, 0.13) | 0.13 (0.1, 0.17) | 0.13 |
| C14:0 | 0.84 (0.75, 0.94) | 1.01 (0.82, 1.19) | 0.118 | 0.94 (0.82, 1.06) | 1.24 (1.06, 1.42) | 0.007 | 0.91 (0.78, 1.05) | 1.15 (1, 1.31) | 0.018 |
| C15:0 | 0.25 (0.24, 0.27) | 0.26 (0.23, 0.29) | 0.598 | 0.25 (0.23, 0.26) | 0.24 (0.22, 0.27) | 0.949 | 0.25 (0.23, 0.28) | 0.24 (0.21, 0.27) | 0.407 |
| C16:0 | 21.53 (21.11, 21.95) | 22.11 (21.33, 22.9) | 0.187 | 21.35 (20.93, 21.78) | 22.03 (21.4, 22.66) | 0.082 | 22.04 (21.35, 22.74) | 22.93 (22.15, 23.7) | 0.08 |
| C17:0 | 0.37 (0.35, 0.39) | 0.36 (0.33, 0.4) | 0.7 | 0.36 (0.34, 0.37) | 0.33 (0.3, 0.35) | 0.044 | 0.32 (0.29, 0.34) | 0.32 (0.3, 0.35) | 0.822 |
| C18:0 | 11.98 (11.71, 12.25) | 11.13 (10.62, 11.64) | 0.004 | 11.83 (11.54, 12.12) | 10.91 (10.48, 11.33) | 0.001 | 11.49 (11.1, 11.88) | 10.94 (10.5, 11.37) | 0.053 |
| C20:0 | 0.09 (0.08, 0.1) | 0.08 (0.07, 0.1) | 0.354 | 0.09 (0.08, 0.1) | 0.11 (0.1, 0.12) | 0.003 | 0.07 (0.07, 0.08) | 0.08 (0.07, 0.09) | 0.294 |
| SFA total | 35.18 (34.7, 35.66) | 35.07 (34.17, 35.97) | 0.826 | 34.94 (34.41, 35.47) | 35.02 (34.24, 35.8) | 0.862 | 35.19 (34.44, 35.94) | 35.79 (34.96, 36.62) | 0.265 |
| C16:1 n7 | 1.31 (1.14, 1.48) | 1.59 (1.26, 1.91) | 0.13 | 1.32 (1.17, 1.48) | 1.57 (1.34, 1.79) | 0.08 | 1.55 (1.33, 1.76) | 1.99 (1.75, 2.23) | 0.005 |
| C18:1 n9 | 19.41 (18.78, 20.05) | 21.11 (19.91, 22.31) | 0.013 | 19.77 (19.09, 20.45) | 23.29 (22.28, 24.31) | <0.001 | 20.16 (19.42, 20.89) | 22.62 (21.8, 23.44) | <0.001 |
| C18:1 c11 | 1.58 (1.5, 1.66) | 1.55 (1.39, 1.71) | 0.728 | 1.51 (1.43, 1.58) | 1.48 (1.37, 1.59) | 0.71 | 1.59 (1.46, 1.71) | 1.53 (1.39, 1.67) | 0.475 |
| C20:1 n9 | 0.28 (0.26, 0.31) | 0.24 (0.19, 0.29) | 0.115 | 0.24 (0.22, 0.25) | 0.26 (0.23, 0.28) | 0.119 | 0.25 (0.23, 0.27) | 0.23 (0.21, 0.25) | 0.073 |
| MUFA total | 22.59 (21.87, 23.3) | 24.49 (23.14, 25.84) | 0.013 | 22.83 (22.07, 23.6) | 26.6 (25.46, 27.74) | <0.001 | 23.55 (22.68, 24.41) | 26.36 (25.4, 27.33) | <0.001 |
| SCD16 | 0.06 (0.05, 0.07) | 0.07 (0.06, 0.08) | 0.128 | 0.06 (0.06, 0.07) | 0.07 (0.06, 0.08) | 0.081 | 0.07 (0.06, 0.08) | 0.09 (0.08, 0.1) | 0.007 |
| SCD18 | 1.63 (1.56, 1.7) | 1.91 (1.77, 2.04) | 0.001 | 1.68 (1.58, 1.78) | 2.19 (2.04, 2.34) | <0.001 | 1.78 (1.67, 1.88) | 2.08 (1.96, 2.2) | <0.001 |
| D5D | 0.01 (0.01, 0.01) | 0.01 (0.01, 0.01) | 0.382 | 0.01 (0.01, 0.01) | 0.01 (0.01, 0.01) | 0.611 | 0.01 (0.01, 0.02) | 0.02 (0.01, 0.02) | 0.324 |
| D6D | 6.31 (5.88, 6.74) | 5.64 (4.83, 6.45) | 0.139 | 5.9 (5.57, 6.22) | 4.78 (4.29, 5.26) | <0.001 | 5.31 (4.88, 5.75) | 5.24 (4.75, 5.73) | 0.811 |
| C18:2 n6 | 19.5 (18.74, 20.26) | 19.57 (18.14, 21) | 0.93 | 20.09 (19.28, 20.89) | 19.74 (18.55, 20.94) | 0.636 | 19.17 (18.16, 20.18) | 17.51 (16.38, 18.64) | 0.026 |
| C18:3 n6 | 0.18 (0.15, 0.2) | 0.2 (0.15, 0.25) | 0.421 | 0.2 (0.18, 0.23) | 0.19 (0.15, 0.23) | 0.617 | 0.25 (0.21, 0.3) | 0.27 (0.21, 0.32) | 0.715 |
| C18:3 n3 | 0.48 (0.43, 0.54) | 0.59 (0.49, 0.7) | 0.056 | 0.49 (0.44, 0.54) | 0.67 (0.6, 0.75) | <0.001 | 0.47 (0.4, 0.54) | 0.54 (0.46, 0.61) | 0.158 |
| C20:3 n6 | 1.26 (1.18, 1.35) | 1.35 (1.19, 1.51) | 0.313 | 1.42 (1.35, 1.5) | 1.37 (1.27, 1.48) | 0.452 | 1.57 (1.45, 1.68) | 1.42 (1.3, 1.55) | 0.078 |
| C20:4 n6 | 7.83 (7.36, 8.31) | 7.51 (6.61, 8.4) | 0.515 | 8.21 (7.89, 8.54) | 6.41 (5.92, 6.89) | <0.001 | 8.28 (7.57, 9) | 7.41 (6.62, 8.21) | 0.094 |
| C20:5 n3 | 1.91 (1.62, 2.19) | 1.08 (0.54, 1.61) | 0.007 | 1.37 (1.12, 1.63) | 1 (0.62, 1.38) | 0.105 | 1.18 (0.98, 1.38) | 1.07 (0.85, 1.29) | 0.442 |
| C22:5 n3 | 1.5 (1.42, 1.58) | 1.26 (1.1, 1.41) | 0.007 | 1.38 (1.3, 1.47) | 1.14 (1.01, 1.26) | 0.002 | 1.29 (1.19, 1.39) | 1.25 (1.14, 1.36) | 0.547 |
| C22:6 n3 | 3.73 (3.47, 3.99) | 3.28 (2.79, 3.78) | 0.111 | 3.31 (3.06, 3.57) | 2.75 (2.37, 3.12) | 0.016 | 3.19 (2.84, 3.55) | 3.01 (2.62, 3.4) | 0.464 |
| PUFA total | 36.39 (35.45, 37.34) | 34.84 (33.06, 36.62) | 0.12 | 36.49 (35.46, 37.51) | 33.27 (31.74, 34.79) | 0.001 | 35.41 (34.19, 36.62) | 32.48 (31.12, 33.83) | 0.001 |
| Omega 3 total | 7.62 (7.08, 8.16) | 6.21 (5.19, 7.23) | 0.015 | 6.56 (6.02, 7.11) | 5.55 (4.74, 6.36) | 0.043 | 6.13 (5.58, 6.69) | 5.87 (5.25, 6.48) | 0.5 |
| Omega 6 total | 28.78 (27.84, 29.71) | 28.63 (26.87, 30.39) | 0.882 | 29.93 (29.06, 30.79) | 27.71 (26.43, 29) | 0.006 | 29.27 (28.16, 30.39) | 26.61 (25.37, 27.85) | 0.002 |

Data are expressed as estimated marginal mean (95 % CI). *P* values from linear regression models adjusted for age and sex. *P* is significant at 0.05 level. SCD1, Stearoyl-CoA desaturase-1; D5D, delta-5-desaturase; D6D, delta-6-desaturase; SFA, saturated fatty acids; MUFA, monounsaturated fatty acids; PUFA, polyunsaturated fatty acids.
