# Supplementary material for: Fatty acid profile and estimated desaturase activities in whole blood are associated with metabolic health
Source: Lipids Health Dis. 2020 May 21;19:102. doi: 10.1186/s12944-020-01282-y (PMC7243306; doi:10.1186/s12944-020-01282-y)
Supplement: Supplementary file 3 — Additional file 3. Association of criteria for metabolic health and estimated desaturase activities in BMI categories. [file 12944_2020_1282_MOESM3_ESM.docx]

**
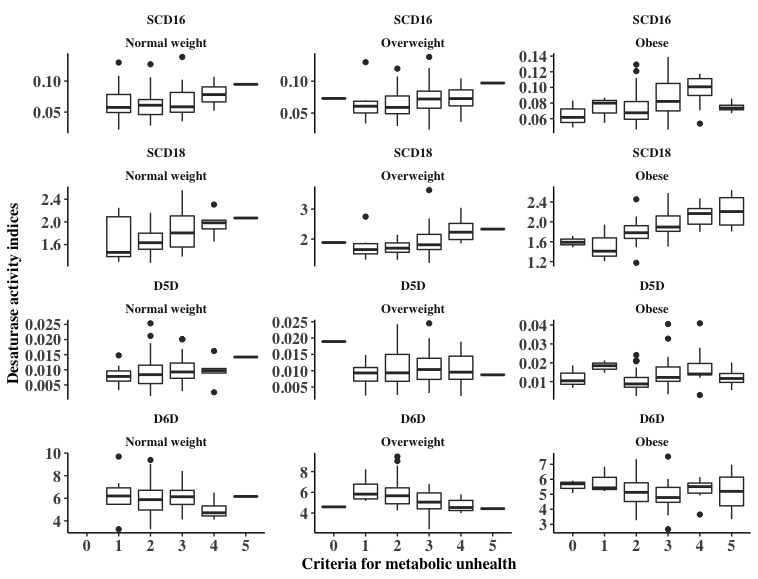
**

**Additional file 3.** Association of criteria for metabolic health and estimated desaturase activities in BMI categories.
