# Supplementary material for: Fatty acid profile and estimated desaturase activities in whole blood are associated with metabolic health
Source: Lipids Health Dis. 2020 May 21;19:102. doi: 10.1186/s12944-020-01282-y (PMC7243306; doi:10.1186/s12944-020-01282-y)
Supplement: Supplementary file 4 — Additional file 4. Diet and lifestyle for metabolically healthy (MH) and unhealthy (MU) participants stratified by body mass index (BMI). [file 12944_2020_1282_MOESM4_ESM.docx]

### **Additional file 4*:*** *Diet and lifestyle for metabolically healthy (MH) and unhealthy (MU) participants stratified by body mass index (BMI).*

|  | Normal weight (BMI 18.5 – 24.9 kg/m^2^) | | | | Overweight (BMI 25 – 29.9 kg/m^2^) | | | | Obese (BMI ≥30 kg/m^2^) | | | |
| --- | --- | --- | --- | --- | --- | --- | --- | --- | --- | --- | --- | --- |
|  | **MH (n=63)** | **MU (n=11)** | ***P*** | **MH (n=52)** | | **MU (n=23)** | ***P*** | **MH (n=34)** | | **MU (n=18)** | ***P*** |  |
| Food (g/day) | median (25^th^, 75^th^ percentiles). | median (25^th^, 75^th^ percentiles). |  | median (25^th^, 75^th^ percentiles). | | median (25^th^, 75^th^ percentiles). |  | median (25^th^, 75^th^ percentiles). | | median (25^th^, 75^th^ percentiles). |  |  |
| Fatty fish | 65.2 (25.4, 92.8) | 33.9 (20.3, 75.2) | 0.166 | 36.9 (20.3, 64.5) | | 45.7 (33.2, 67.5) | 0.073 | 43.9 (14.7, 71.7) | | 42.3 (3.9, 76.5) | 0.630 |  |
| Lean fish | 30.4 (20.3, 42.1) | 20.3 (5.0, 52.1) | 0.160 | 20.3 (10.1, 49.6) | | 40.6 (20.3, 62.9) | 0.188 | 30.4 (10.1, 41.0) | | 20.3 (11.4, 52.1) | 0.892 |  |
| Total fish | 92.8 (63.5, 141) | 80.2 (38.9, 112) | 0.149 | 66.3 (41.2, 106) | | 96.1 (53.5, 125) | 0.067 | 73.9 (43.2, 107) | | 79.7 (31.2, 104) | 0.939 |  |
| All products with whole grain | 97.6 (59.2, 142) | 109 (54.0, 167) | 0.958 | 77.7 (40.4, 120) | | 114 (61.0, 143) | 0.039 | 99.3 (49.1, 144) | | 79.0 (59.6, 137) | 0.700 |  |
| Products with high content of SFA | 82.3 (50.4, 158) | 67.1 (17.8, 112) | 0.057 | 84.2 (55.5, 130) | | 112 (66.6, 167) | 0.215 | 87.2 (55.2, 115) | | 67.2 (41.4, 212) | 0.901 |  |
| Products with high content of sugar | 51.5 (28.5, 80.5) | 35.8 (12.9, 63.4) | 0.134 | 55.4 (26.7, 96.6) | | 46.6 (19.0, 93.8) | 0.743 | 39.9 (10.7, 67.1) | | 60.5 (28.0, 99.9) | 0.104 |  |
| Alcohol (g/day) |  |  |  |  | |  |  |  | |  |  |  |
| Total drinks containing alcohol | 63.8 (7.7, 158) | 14.2 (0, 125) | 0.177 | 68.3 (0, 200) | | 30.8 (0, 156) | 0.661 | 37.3 (0, 127) | | 16.0 (0, 181) | 0.751 |  |
|  |  |  |  |  | |  |  |  | |  |  |  |
| Physical activity (min/week) | 247 (117, 508) | 152 (0, 319) | 0.117 | 218 (102, 469) | | 160 (90, 393) | 0.369 | 150 (0, 362) | | 38 (0, 126) | 0.031 |  |

Data are expressed as median (25^th^, 75^th^ percentiles). P values from Mann-Whitney test. *P* is significant at 0.05 level and highlighted in bold.

MH when fulfilling ≥ three of the following: total cholesterol ≥ 5.2 mmol/L, LDL-C ≥ 2.6 mmol/L, HDL-C ≤ 1.3 mmol/L, triglycerides ≥ 1.7 mmol/L (fasting) or ≥ 2.1 mmol/L (non-fasting) or HbA1c ≥ 5.7%. MU when fulfilling ≥ four of the following: total cholesterol < 5.2 mmol/L, LDL-C < 2.6 mmol/L, HDL-C > 1.3 mmol/L, triglycerides < 1.7 mmol/L (fasting) or < 2.1 mmol/L (non-fasting) or HbA1c < 5.7%.

SFA, saturated fatty acids.
